# Relationship of Immune-Related Adverse Events with Tumor Response and Prognosis in Esophageal Squamous Cell Carcinoma Following Nivolumab Monotherapy

**DOI:** 10.3390/cancers16203529

**Published:** 2024-10-18

**Authors:** Yoichi Hamai, Yuta Ibuki, Tomoaki Kurokawa, Ryosuke Hirohata, Manato Ohsawa, Nao Kitasaki, Manabu Emi, Morihito Okada

**Affiliations:** Department of Surgical Oncology, Hiroshima University, Hiroshima 734-8551, Japan; ahryyibuki@gmail.com (Y.I.); tomokanakurokawa@yahoo.co.jp (T.K.); ryocobain88627@gmail.com (R.H.); ohsawa311@gmail.com (M.O.); tennis.xylitol@gmail.com (N.K.); moaista@hotmail.com (M.E.); morihito1217@gmail.com (M.O.)

**Keywords:** adverse event, cancer, esophagus, immunotherapy, nivolumab, response, survival

## Abstract

Background: Patients across various cancers who develop immune-related adverse events (irAEs) post-immune checkpoint inhibitor (ICI) treatment tend to experience better tumor response and survival than those who do not. However, studies regarding this association in patients with esophageal squamous cell carcinoma (ESCC) are limited. Methods: We assessed the relationship of irAEs with tumor response and survival in 82 consecutive patients with unresectable advanced or recurrent ESCC treated with second- or later-line nivolumab, an anti-PD-1 antibody, monotherapy. Results: We observed irAEs in 24 (29.3%) patients, with the overall response and disease control rates in the irAE-positive group being significantly better than those in the irAE-negative group (both *p* < 0.0001). During the entire period and within 8 weeks of nivolumab initiation, progression-free and overall survivals (PFS and OS, respectively) were significantly better in patients with grade1/2 irAEs than in those without. Univariate and multivariate analyses indicated grade1/2 irAEs during the entire period and within 8 weeks as independent covariates for PFS (entire period: hazard ratio [HR] 0.28, 95% confidence interval [CI] 0.16–0.49, *p* < 0.001; within 8 weeks: HR 0.46, 95% CI 0.23–0.93, *p* = 0.03) and OS (entire period: HR 0.24, 95% CI 0.13–0.44, *p* < 0.001; within 8 weeks: HR 0.41, 95% CI 0.18–0.92, *p* = 0.03). Conclusions: Grade1/2 irAEs during the entire treatment period and within 8 weeks of nivolumab initiation were significantly associated with improved tumor response and survival in patients with advanced ESCC treated with nivolumab monotherapy. Therefore, mild irAEs may be predictive markers for the response and prognosis of ESCC following ICI treatment.

## Simple Summary

Previous studies have demonstrated that the occurrence of immune-related adverse events (irAEs) is significantly associated with favorable efficacy and prognosis in patients with cancers treated with immune checkpoint inhibitors (ICIs), such as anti-programmed cell death protein 1 (PD-1) and anti-cytotoxic T lymphocyte-associated antigen. However, few studies have investigated this association in patients with esophageal squamous cell carcinoma (ESCC). Herein, we evaluated the relationship of irAEs with tumor response and survival in patients with unresectable advanced or recurrent ESCC treated with second- or later-line nivolumab, an anti-PD-1 antibody, monotherapy. We observed that the occurrence of mild (grade 1/2) irAEs during the entire period, as well as within 8 weeks of nivolumab initiation, was significantly associated with tumor response and survival following nivolumab monotherapy. Thus, mild irAEs may serve as predictive markers for the response and prognosis of patients with ESCC treated with ICIs.

## 1. Introduction

Recently, various immune checkpoint inhibitors (ICIs) have been reported to demonstrate antitumor efficacy and clinically significant benefits in patients with esophageal cancer [1]. Nivolumab, an anti-PD-1 antibody, monotherapy as the second-line treatment significantly improves the survival of patients with unresectable advanced or recurrent esophageal squamous cell carcinoma (ESCC) [2,3]. Furthermore, nivolumab as the first-line treatment [4] and postoperative adjuvant therapy [5] significantly improves the survival of patients with advanced ESCC. Therefore, nivolumab is currently a key drug for the treatment of advanced ESCC.

ICIs can cause immune-related adverse events (irAEs) in some patients by enhancing the cellular and humoral immune responses, which differ considerably from AEs caused by conventional chemotherapy or target drugs [6]. Although the rates of irAEs have generally remained low and acceptable as demonstrated in clinical trials [1,2,3,4,5], they can affect any organ and range from mild side effects to life-threatening complications on rare occasions [7,8].

The detailed mechanisms underlying irAEs remain unclear; however, the enhancement of systemic T-cell activity by ICIs—leading to a loss of immune tolerance in various organs—is considered to manifest in irAEs. Given that irAEs and improved clinical outcomes share a similar immunological basis, a possible association between the two is hypothesized to exist [9,10,11]. In various cancers, patients who develop irAEs experience greater clinical benefits compared with those who do not [12]. However, few studies have investigated this relationship in patients with ESCC. Therefore, we evaluated the relationship of irAEs with tumor response and survival in patients with unresectable advanced or recurrent ESCC treated with second- or later-line nivolumab monotherapy.

## 2. Materials and Methods

### 2.1. Patients

We retrospectively reviewed data from 83 consecutive patients with unresectable advanced or recurrent ESCC who received nivolumab monotherapy after at least one prior chemotherapeutic regimen at our institution between June 2016 and August 2023, of which one patient with concurrent advanced rectal cancer was excluded. Finally, the association of irAEs with tumor response and survival was assessed in 82 patients.

Histological tumors were diagnosed as ESCC based on biopsy samples obtained be-fore treatment for unresectable advanced patients or from resected specimens for recurrent patients. Before initiating nivolumab therapy, blood tests, including endocrine function tests, were performed to evaluate the functional parameters of vital organs and the presence or absence of endocrine disorder, and chest X-rays, electrocardiograms, and computed tomography (CT) scans were used for image assessment of each organ. The surgeons, oncologists, and radiologists of our institution determined the therapeutic strategy for nivolumab administration for each patient after a discussion.

This study was approved by the Institutional Review Board of Hiroshima University (approval no. E-2225) and was conducted in compliance with the Declaration of Helsinki (2013). The requirement for informed consent was waived because this study was a retrospective review of a patient database, and all data were anonymized and presented in aggregates.

### 2.2. Nivolumab Therapy and Clinical Response Assessment

Among the included 82 patients, 59 and 23 were administered 240 mg and 480 mg intravenous nivolumab at 2- and 4-week intervals, respectively. Both administration methods were based on the prescribed regimen.

Clinical response to nivolumab was evaluated in all patients using CT scan every 2 months. Responses were evaluated earlier for patients with large tumor volumes or poor general condition. Patients with worsening symptoms were examined at the discretion of the attending physician. All patients had measurable target locations such as lymph nodes and/or organ metastases. Clinical tumor responses to nivolumab were judged as complete response (CR), partial response (PR), stable disease (SD), and progressive disease (PD) according to the Response Evaluation Criteria in Solid Tumors (RECIST) [13].

### 2.3. irAEs

Data regarding patient information and irAEs were collected from electronic medical charts and our patient database. All irAEs were graded according to the Common Terminology Criteria for Adverse Events version 5.0 [14]. Grades refer to the severity of the AEs, which were graded from 1 to 5 with respect to these criteria (grade 1: mild; 2: moderate; 3: severe; 4: life-threating; 5: death).

Cases without irAEs were classified as grade 0. We also separated irAEs according to grades of severity and defined grade 1/2 and ≥3 as mild and severe irAEs, respectively, for the present study. Furthermore, we defined irAEs occurring within 8 weeks of nivolumab initiation as early-period irAEs to assess the association between irAEs occurring in the early period and clinical outcomes.

### 2.4. Statistical Analyses

Categorical variables were analyzed using χ^2^ tests, and continuous variables were analyzed using unpaired *t*-tests. Survival outcomes were evaluated in August 2024. Progression-free survival (PFS) was defined as the interval from nivolumab initiation until the first event (tumor progression or death from any cause) or the most recent follow-up. Overall survival (OS) was defined as the interval from nivolumab initiation to death due to any cause or the most recent follow-up. Survival data were analyzed using Kaplan–Meier curves and compared using log-rank tests.

We evaluated the effects of various clinicopathological factors and irAEs on survival using univariate analyses and determined independent predictors of survival using multivariate Cox proportional hazard analyses with backward elimination. Statistical significance was set at *p* < 0.05. All data were analyzed using SPSS version 27 (IBM Corp., Armonk, NY, USA).

## 3. Results

### 3.1. Patient Characteristics

Table 1 shows the clinical characteristics of patients at the time of nivolumab initiation. The irAEs were observed in 24 patients (29.3%) during the study period. Nivolumab regimens of 240 mg every 2 weeks and 480 mg every 4 weeks resulted in irAEs in 18 and 6 patients, respectively. No significant differences in any characteristics or in each regimen were observed between the irAE-positive and irAE-negative groups (n = 24 and 58, respectively).

### 3.2. irAEs

Table 2 shows the grades and characteristics of irAEs observed in 24 patients (29.3%) during the study period. irAEs of grades 1, 2, and 3 were observed in 5 (6.1%), 17 (20.7%), and 2 (2.4%) patients, respectively; none of the patients experienced grade 4 or 5 irAEs. Four and three patients experienced two and three irAEs, respectively. Therefore, the total number of irAEs was 31. The median time from nivolumab initiation to the occurrence of irAEs was 60 (14–427) days. Two patients developed grade 3 irAEs on post-nivolumab initiation days 27 and 129.

The most common irAEs were hypothyroidism and rash in eight patients (9.8%), followed by pruritus in five patients (6.1%), drug-induced pneumonitis, diarrhea, and hepatic disorder in three patients (3.7%), and adrenal insufficiency, pancreatitis, enterocolitis, and renal disorder in one (1.2%) patient.

### 3.3. Comparison of Tumor Responses According to irAEs

Table 3 shows tumor responses to nivolumab in all patients and comparisons of tumor responses between the irAEs-positive and -negative groups. The best clinical responses of all patients were CR in 2 (2.4%), PR in 16 (19.5%), SD in 21 (25.6%), and PD in 43 (52.4%) patients. Therefore, the objective response rate (ORR [CR + PR]) and disease control rate (DCR [CR + PR + SD]) were 22.0 and 47.6%, respectively.

Furthermore, tumor responses were separately assessed in two regimens of nivolumab. The best clinical responses of patients who were administered nivolumab with 240 mg every 2 weeks (n = 59) were CR in 1 (1.7%), PR in 11 (18.6%), SD in 17 (28.8%), and PD in 30 (50.8%) patients. The best clinical responses of patients who were administered nivolumab with 480 mg every 4 weeks (n = 23) were CR in 1 (4.3%), PR in 5 (21.7%), SD in 4 (17.4%), and PD in 13 (56.5%) patients. There are no significant differences in tumor responses between each regimen (*p* = 0.68).

For the entire period, the best clinical responses were CR in 2 (8.3%), PR in 12 (50.0%), SD in 6 (25.0%), and PD in 4 (16.7%) patients with irAEs and CR in 0 (0%), PR in 4 (6.9%), SD in 15 (25.9%), and PD in 39 (67.2%) patients without irAEs. Therefore, the ORR and DCR in the irAE-positive group were significantly better than those in the irAE-negative group (both *p* < 0.0001).

Furthermore, we compared the tumor responses between irAEs-positive and negative groups within 8 weeks from nivolumab initiation to assess the association of tumor responses and early-period irAEs. Twelve patients developed irAEs within 8 weeks of initiating nivolumab therapy. For the 8-week period, the ORR and DCR were significantly higher in the irAEs-positive group than in the irAE-negative group (*p* = 0.01 and 0.04, respectively).

### 3.4. Survivals of the Patients Treated with Nivolumab

**Figure 1:**
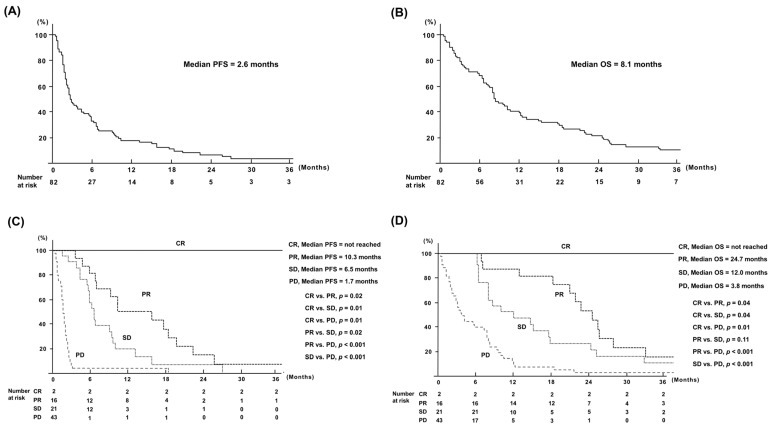
Survival rates of all patients treated by nivolumab: (**A**) PFS of all patients; (**B**) OS of all patients; (**C**) PFS according to tumor responses; (**D**) OS according to tumor responses. OS, overall survival; PFS, progression-free survival. PFS, progression-free survival; OS, overall survival; CR, complete response; PR, partial response; SD, stable disease; PD, progressive disease.

The median PFS and OS of all patients were 2.6 and 8.1 months, respectively (Figure 1A,B). Furthermore, survival was evaluated according to clinical tumor response. Two patients maintained CR without tumor progression for more than 45 months after initiating nivolumab therapy. The median PFS of patients with PR, SD and PD were 10.3, 6.5, and 1.7 months, respectively (CR vs. PR, *p* = 0.02; CR vs. SD, *p* = 0.01; CR vs. PD, *p* = 0.01; PR vs. SD, *p* = 0.02; PR vs. PD, *p* < 0.001; SD vs. PD, *p* < 0.001; Figure 1C). The median OS of patients with PR, SD, and PD were 24.7, 12.0, and 3.8 months, respectively (CR vs. PR, *p* = 0.04; CR vs. SD, *p* = 0.04; CR vs. PD, *p* = 0.01; PR vs. SD, *p* = 0.11; PR vs. PD, *p* < 0.001; SD vs. PD, *p* < 0.001; Figure 1D).

### 3.5. Survivals According to irAEs

**Figure 2:**
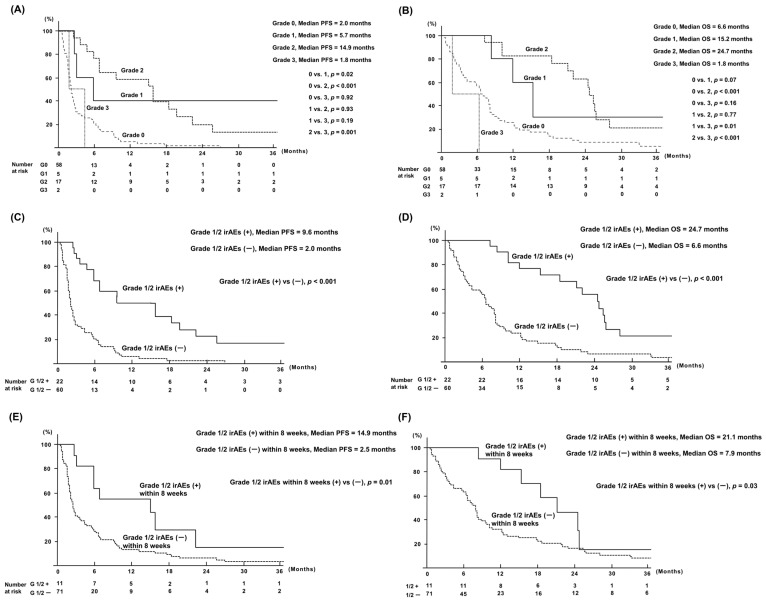
Survival rates according to irAEs: (**A**) PFS of all patients according to irAE grades; (**B**) OS of all patients according to irAE grades; (**C**) PFS of patients with and without grades 1/2 irAEs during the entire period of nivolumab monotherapy; (**D**) OS of patients with and without grades 1/2 irAEs during the entire period of nivolumab monotherapy; (**E**) PFS of patients with and without grades 1/2 irAEs during 8 weeks from nivolumab monotherapy initiation; (**F**) OS of patients with and without grades 1/2 irAEs during 8 weeks from nivolumab monotherapy initiation. OS, overall survival; PFS, progression-free survival; irAEs, immune-related adverse events. irAEs, immune-related adverse events; PFS, progression-free survival; OS, overall survival.

Survival was evaluated according to irAE grades (Figure 2). The median PFS of patients with grade 0, 1, 2, and 3 irAEs were 2.0, 5.7, 14.9, and 1.8 months, respectively (grade 0 vs. 1, *p* = 0.02; 0 vs. 2, *p* < 0.001; 0 vs. 3, *p* = 0.92; 1 vs. 2, *p* = 0.93; 1 vs. 3, *p* = 0.19; 2 vs. 3, *p* = 0.001; Figure 2A). The median OS of patients with grade 0, 1, 2, and 3 irAEs were 6.6, 15.2, 24.7, and 1.8 months, respectively (grade 0 vs. 1, *p* = 0.07; 0 vs. 2, *p* < 0.001; 0 vs. 3, *p* = 0.16; 1 vs. 2, *p* = 0.77; 1 vs. 3, *p* = 0.01; 2 vs. 3, *p* < 0.001; Figure 2B). The median PFS and OS of patients with grade 3 irAEs were poorer than those of patients without irAEs (grade 0) and those with grade 1 or 2 irAEs. PFS and OS of patients with grade 3 irAEs were significantly worse than those of patients with grade 1 or 2 irAEs.

Accordingly, we considered grade 1 and 2 irAEs (n = 22) to be favorable prognostic factors for patients treated with nivolumab monotherapy and compared their survival rates with those of patients without grade1/2 irAEs (patients with grade 0 or 3 irAEs, n = 60). The median PFS of patients with and without grade1/2 irAEs were 9.6 and 2.0 months, respectively (*p* < 0.001; Figure 2C). The median OS of patients with and without grade1/2 irAEs were 24.7 and 6.6 months, respectively (*p* < 0.001; Figure 2D). PFS and OS differed significantly between patients with and without grade1/2 irAEs.

Furthermore, we evaluated the differences in survival between patients with and without grade1/2 irAEs within 8 weeks (n = 11 and n = 71, respectively) to assess the association between early-period irAEs and survival outcomes. The median PFS of patients with and without grade1/2 irAEs within 8 weeks were 14.9 and 2.5 months, respectively (*p* = 0.01; Figure 2E). The median OS of patients with and without grade1/2 irAEs within 8 weeks were 21.1 and 7.9 months, respectively (*p* = 0.03; Figure 2F). PFS and OS differed significantly between patients with and without grade1/2 irAEs within 8 weeks.

### 3.6. Univariate and Multivariate Analyses of Survival Predictors

Univariate analysis showed that performance status (PS) and grade1/2 irAEs during the entire period and within 8 weeks were significant prognostic factors associated with PFS (Table 4). Subsequently, independent factors influencing the PFS were analyzed using multivariate analyses that separately included either grade1/2 irAEs during the entire period or within 8 weeks, along with other factors, to avoid confounding (multivariate 1 and 2, respectively). Multivariate analyses identified both grade1/2 irAEs during the entire period and within 8 weeks as significant independent covariates for PFS (multivariate 1: irAEs during the entire period, hazard ratio [HR] 0.28, 95% confidence interval [CI] 0.16–0.49, *p* < 0.001; multivariate 2: irAEs within 8 weeks, HR 0.46, 95% CI 0.23–0.93, *p* = 0.03).

Similarly, univariate and multivariate analyses for OS (Table 5) identified both grade1/2 irAEs during the entire period and within 8 weeks as significant independent covariates for OS (multivariate 1: grade1/2 irAEs during the entire period, HR 0.24, 95% CI 0.13–0.44, *p* < 0.001; multivariate 2: grade1/2 irAEs within 8 weeks, HR 0.41, 95% CI 0.18–0.92, *p* = 0.03).

## 4. Discussion

The number of patients with ESCC undergoing ICI treatment is on the rise. The use of nivolumab in multiple therapeutic settings—postoperative adjuvant therapy, as well as first- and second-line therapies for unresectable advanced or recurrent esophageal tumors—has significantly improved survival outcomes in ESCC patients [1,2,3,4,5]. Herein, we evaluated the association between irAEs and tumor response and prognosis in patients with unresectable advanced or recurrent ESCC who underwent second- or later-line nivolumab monotherapy. The survival was significantly longer in patients with grade 1 or 2 irAEs than in those without. The occurrence of grade1/2 irAEs, not only during the entire period but also within 8 weeks, was significantly associated with tumor responses and prognoses in patients with ESCC treated with nivolumab monotherapy.

Herein, the ORR and DCR were 22 and 47.6%, respectively, and median PFS and OS were 2.6 and 8.1 months, respectively. It was shown in the ATTRACTION-3 trial that the ORR and DCR were 22.4 and 41.1%, respectively, and the median PFS and OS were 2.7 and 13.4 months, respectively, in the Japanese subpopulation [3]. In the present study, irAEs were observed in 24 patients (29.3%). The most common irAEs were hypothyroidism and rash (9.8%); others included pruritus (6.1%), drug-induced pneumonitis, diarrhea, and hepatic disorder (3.7%), and adrenal insufficiency, pancreatitis, enterocolitis, and renal disorder (1.2%). In the aforementioned cohort, hypothyroidism, rash, and diarrhea were reported in 10, 11, and 7% of patients, respectively [3]. Therefore, the OS was relatively poorer in the present study population than in the Japanese subpopulation of the ATTRACTION-3 trial [3]. This was probably because our study enrolled patients with diverse clinical profiles who did not meet the trial eligibility criteria, including those with poor PS, comorbidities, or multiple treatments. However, clinical tumor response, PFS, and frequency of irAEs did not differ substantially between these two studies [3].

Previous reports suggest that the occurrence of irAEs is significantly associated with favorable tumor response and prognosis in various cancers treated with ICIs [15,16,17,18]. A few reports on esophageal cancer have demonstrated the correlation between irAEs and treatment efficacy of ICIs, and the potential for irAEs to predict treatment efficacy in patients with esophageal cancer undergoing ICI therapy [19,20,21,22]. However, these reports included patients with gastric cancer and adenocarcinoma of the esophagus, as well as patients who underwent various ICI monotherapies, combined chemotherapy, targeted medicine, or radiotherapy [19,20,21,22]. Herein, we evaluated the association of irAEs with treatment efficacy and prognosis in a homogeneous cohort of patients with ESCC treated with nivolumab monotherapy.

The main mechanism of irAEs is considered to be the destruction of autologous cells and tissues by autoantibodies and the accidental activation of autoantigen-specific lymphocytes, which are produced and remain in the body without being removed following the administration of ICIs [23,24]. However, it is difficult to characterize irAEs definitively through a singular mechanism due to the diversity in cancer types, affected organs, and individual patients. [8,23,24]. Furthermore, severe irAEs are typically treated by interrupting or permanently discontinuing ICI treatment and administering empirically selected systemic immunosuppressive agents [8], which can diminish the antitumor effects of ICIs. Therefore, while clinical observations suggest that low-grade irAEs are positively associated with responses to ICIs, the correlation between severe irAEs and clinical benefits is less clear [8,15,25,26].

We found that irAE severity was significantly associated with RFS and OS after nivolumab monotherapy. The survival of patients with grade 3 irAEs was much poorer than that of patients with grade 1 or 2 irAEs and were relatively similar to that of the patients without irAEs; therefore, we considered grade1/2 irAEs to be better prognostic indicators. Conversely, our findings suggest that early detection and rapid treatment of irAEs before they become progressively more severe are needed to further improve the outcomes for patients treated with ICIs because severe irAEs are directly associated with poorer survival.

The median time from the initiation of nivolumab to the occurrence of irAEs was 60 (14–427) days in 24 patients, half of whom developed irAEs within 8 weeks of nivolumab initiation. Patients who develop irAEs in the late period should have favorable clinical outcomes because nivolumab could continue the clinically effective response and be used for a long period. Therefore, we evaluated tumor responses and prognoses in patients with irAEs not only during the entire period but also within 8 weeks of nivolumab initiation. Tumor response and survival were better in patients with grade1/2 irAEs within 8 weeks of nivolumab initiation than in other patients; mild irAEs in the early period were significantly associated with the efficacy of nivolumab and subsequent survival of patients.

Our study has several limitations. First, this was a single-center retrospective study. Second, our study included some patients who underwent nivolumab as a later than second-line therapy. Additionally, two regimens of nivolumab therapy were used, administered at 240 and 480 mg at 2- and 4-weeks intervals, respectively. This might have affected the occurrence of irAEs to some extent after nivolumab therapy. However, our findings are important for understanding the association between irAEs and ICI efficacy, and survival in patients with unresectable advanced or recurrent ESCC, which may aid the management of such patients. While we assessed the correlation between irAEs and tumor response as well as prognosis in a homogeneous cohort of patients with ESCC receiving solely nivolumab monotherapy, additional investigations involving other ICIs and diverse combination therapies incorporating ICIs are necessary to acquire an in-depth understanding of the association between irAEs, tumor response, and survival.

## 5. Conclusions

The occurrence of grade1/2 irAEs, not only during the entire period but also within 8 weeks, was significantly associated with tumor response and survival in patients with advanced ESCC treated with nivolumab monotherapy. Mild (grade 1/2) irAEs may serve as predictive markers of tumor response and prognosis in patients with ESCC treated with nivolumab. Early detection and rapid treatment of irAEs before they become progressively more severe are required to further improve the outcomes of patients treated with ICIs.

## Figures and Tables

**Table 1:** Patient characteristics and comparison of characteristics between the irAE-positive and -negative groups.

Parameters	All Patients n = 82	irAEs (+) n = 24	irAEs (−) n = 58	*p*-Value
Age (years)	70.0 ± 8.7	70.6 ± 7.9	69.7 ± 9.0	0.68
Sex				
Men	70 (85.4)	19 (79.2)	51 (87.9)	0.31
Women	12 (14.6)	5 (20.8)	7 (12.1)	
Performance status *				
0	38 (46.3)	15 (62.5)	23 (39.7)	0.12
1	34 (41.5)	8 (33.3)	26 (44.8)	
≥2	10 (12.2)	1 (4.2)	9 (15.5)	
Body mass index (kg/m^2^)	19.4 ± 3.5	20.1 ± 3.9	19.0 ± 3.4	0.20
Smoking history				
+	68 (82.9)	18 (75.0)	50 (86.2)	0.22
−	14 (17.1)	6 (25.0)	8 (13.8)	
Brinkman index **	587 ± 488	684 ± 679	547 ± 382	0.25
Alcohol history				
+	72 (87.8)	20 (83.3)	52 (89.7)	0.43
−	10 (12.2)	4 (16.7)	6 (10.3)	
Primary tumor location				
Cervical	17 (20.7)	6 (25.0)	11 (19.0)	0.79
Upper and Middle third	45 (54.9)	13 (54.2)	32 (55.2)	
Lower third and EGJ	20 (24.4)	5 (20.8)	15 (25.9)	
Histological type				
Well differentiated	7 (8.5)	0 (0)	7 (12.1)	0.17
Moderately differentiated	35 (42.7)	12 (50.0)	23 (39.7)	
Poorly differentiated	24 (29.3)	9 (37.5)	15 (25.9)	
Not assessable	16 (19.5)	3 (12.5)	13 (22.4)	
Diseases status				
Postoperative recurrence	34 (41.5)	8 (33.3)	26 (44.8)	0.34
Unresectable advanced	48 (58.5)	16 (66.7)	32 (55.2)	
Number of previous chemotherapies ***				
1	68 (82.9)	18 (75.0)	50 (86.2)	0.22
≥2	14 (17.1)	6 (25.0)	8 (13.8)	
Number of organs with metastasis ****				
1	34 (41.5)	12 (50.0)	22 (37.9)	0.57
2	30 (36.6)	8 (33.3)	22 (37.9)	
≥3	18 (22.0)	4 (16.7)	14 (24.1)	
Regimen of nivolumab				
240 mg every 2 weeks	59 (72.0)	18 (75.0)	41 (70.7)	0.69
480 mg every 4 weeks	23 (28.0)	6 (25.0)	17 (19.3)	

All values except that for age, body mass index and brinkman index are shown as n (%). Age, body mass index and brinkman index are shown as mean ± standard deviation. EGJ, esophagogastric junction; ESCC, esophageal squamous cell carcinoma; irAEs, immune-related adverse events. * Eastern Cooperative Oncology Group performance status. ** The number of cigarettes per day × the number of smoking years. *** Number of treatment regimens from the diagnosis of unresectable advanced or recurrent ESCC until nivolumab. **** At the time of nivolumab initiation.

**Table 2:** Immune-related adverse events.

Worst Grade of irAEs * (n = 82)			
Grade 0 **	58 (70.7%)		
Grade 1	5 (6.1%)		
Grade 2	17 (20.7%)		
Grade 3	2 (2.4%)		
Grade 4/5	0		
irAEs *** (n = 31)	Grade 1 (n = 9)	Grade 2 (n = 23)	Grade 3 (n = 2)
Hypothyroidism (n = 8)	0	8	0
Rash (n = 8)	3	5	0
Pruritus (n = 5)	3	2	0
Drug-induced pneumonitis (n = 3)	0	2	1
Diarrhea (n = 3)	1	2	0
Hepatic disorder **** (n = 3)	2	1	0
Adrenal insufficiency (n = 1)	0	0	1
Pancreatitis (n = 1)	0	1	0
Enterocolitis (n = 1)	0	1	0
Renal disorder ***** (n = 1)	0	1	0

irAEs, immune-related adverse events. * According to the Common Terminology Criteria for Adverse Events version 5.0. ** The cases without irAEs were represented as Grade 0. *** There were four and three cases with two and three irAEs, respectively. **** Alanine aminotransferase and/or aspartate aminotransferase increased. ***** Creatinine increased.

**Table 3:** Tumor responses for all patients and for patients with and without immune-related adverse events.

All Patients (n = 82)			
Best clinical response *			
CR	2 (2.4)		
PR	16 (19.5)		
SD	21 (25.6)		
PD	43 (52.4)		
Objective response **	18 (22.0)		
Disease control ***	39 (47.6)		
irAEs during the entire period			
Best clinical response *	irAEs (+) n = 24	irAEs (−) n = 58	*p*-value ****
CR	2 (8.3)	0 (0)	<0.0001
PR	12 (50.0)	4 (6.9)	
SD	6 (25.0)	15 (25.9)	
PD	4 (16.7)	39 (67.2)	
Objective response **	14 (58.3)	4 (6.9)	<0.0001
Disease control ***	20 (83.3)	19 (32.8)	<0.0001
irAEs within 8 weeks			
Best clinical response *	irAEs within 8 weeks (+) n = 12	irAEs within 8 weeks (−) n = 70	*p*-value ****
CR	0 (0)	2 (2.9)	0.03
PR	6 (50.0)	10 (14.3)	
SD	3 (25.0)	18 (25.7)	
PD	3 (25.0)	40 (57.1)	
Objective response **	6 (50.0)	12 (17.1)	0.01
Disease control ***	9 (75.0)	30 (42.9)	0.04

All values are shown as n (%). CR, complete response; irAE, immune-related adverse events; PD, progressive disease; PR, partial response; SD, stable disease. * According to the Response Evaluation Criteria in Solid Tumors. ** Objective response: CR + PR. *** Disease control: CR + PR + SD. **** Analyzed using χ^2^ tests.

**Table 4:** Univariate and multivariate analyses for progression-free survival.

	Univariate			Multivariate 1 ****			Multivariate 2 ****		
Variables	HR	95% CI	*p*-Value	HR	95% CI	*p*-Value	HR	95% CI	*p*-Value
Age (mean ± SD, y)	1.005	0.98–1.03	0.72	-	-	-	-	-	-
Sex									
Female	1			-	-	-	-	-	-
Male	1.37	0.72–2.63	0.34	-	-	-	-	-	-
Performance status *									
0	1			1			1		
≥1	1.96	1.24–3.09	0.004	1.95	1.22–3.13	0.01	1.85	1.17–2.92	0.01
Body mass index (kg/m^2^) (continuous)	0.97	0.91–1.04	0.38	-	-	-	-	-	-
Smoking history									
−	1			-	-	-	-	-	-
+	1.12	0.62–2.17	0.65	-	-	-	-	-	-
Primary tumor location									
Cervical and upper third	1			-	-	-	-	-	-
Middle and lower third and EGJ	1.05	0.65–1.71	0.83	-	-	-	-	-	-
Diseases status									
Unresectable advanced	1			-	-	-	-	-	-
Postoperative recurrence	0.97	0.62–1.52	0.90	-	-	-	-	-	-
Histological type									
Poorly differentiated	1			-	-	-	-	-	-
Others	0.97	0.60–1.59	0.91	-	-	-	-	-	-
Number of previous chemotherapies **									
1	1			-	-	-	-	-	-
≥2	0.62	0.35–1.11	0.11	-	-	-	-	-	-
Number of organs with metastasis ***									
1	1			-	-	-	-	-	-
≥2	1.07	0.68–1.70	0.76	-	-	-	-	-	-
Regimen of nivolumab									
240 mg every 2 weeks	1			-	-	-	-	-	-
480 mg every 4 weeks	1.09	0.84–1.40	0.52	-	-	-	-	-	-
Grade1/2 irAEs during entire period									
−	1			1			-	-	-
+	0.28	0.16–0.49	<0.001	0.28	0.16–0.49	<0.001	-	-	-
Grade1/2 irAEs within 8 weeks									
−	1			-	-	-	1		
+	0.43	0.21–0.86	0.02	-	-	-	0.46	0.23–0.93	0.03

EGJ, esophagogastric junction; irAE, immune-related adverse events; CI, confidence interval; HR, hazard ratio. * Eastern Cooperative Oncology Group performance status. ** Number of treatment regimens from the diagnosis of unresectable advanced or recurrent ESCC until nivolumab. *** At the time of nivolumab initiation. **** Backward elimination method.

**Table 5:** Univariate and multivariate analyses for overall survival.

	Univariate			Multivariate 1 ****			Multivariate 2 ****		
Variables	HR	95% CI	*p*-Value	HR	95% CI	*p*-Value	HR	95% CI	*p*-Value
Age (mean ± SD, y)	1.01	0.99–1.04	0.30	-	-	-	-	-	-
Sex									
Women	1			-	-	-	-	-	-
Men	1.20	0.62–2.35	0.59	-	-	-	-	-	-
Performance status *									
0	1			1			1		
≥1	2.89	1.78–4.68	<0.001	3.53	2.09–5.95	<0.001	3.62	2.02–6.49	<0.001
Body mass index (kg/m^2^) (continuous)	0.97	0.90–1.04	0.36	-	-	-	-	-	-
Smoking history									
−	1			-	-	-	-	-	-
+	1.16	0.61–2.21	0.66	-	-	-	-	-	-
Primary tumor location									
Cervical and upper third	1			-	-	-	-	-	-
Middle and lower third and EGJ	1.001	0.61–1.63	0.998	-	-	-	-	-	-
Diseases status									
Postoperative recurrence	1			-	-	-	-	-	-
Unresectable advanced	1.09	0.69–1.75	0.71	-	-	-	-	-	-
Histological type									
Poorly differentiated	1			-	-	-	-	-	-
Others	0.74	0.44–1.25	0.26	-	-	-	-	-	-
Number of previous chemotherapies **									
1	1			-	-	-	-	-	-
≥2	0.46	0.24–0.88	0.02	-	-	-	-	-	-
Number of organs with metastasis ***									
1	1			-	-	-	-	-	-
≥2	1.15	0.71–1.85	0.57	-	-	-	-	-	-
Regimen of nivolumab									
240 mg every 2 weeks	1			-	-	-	-	-	-
480 mg every 4 weeks	1.10	0.85–1.43	0.46	-	-	-	-	-	-
irAE (G1/2) during entire period									
−	1			1			-	-	-
+	0.29	0.16–0.51	<0.001	0.24	0.13–0.44	<0.001	-	-	-
irAE (G1/2) within 8 weeks									
−	1			-	-	-	1		
+	0.42	0.19–0.92	0.03	-	-	-	0.41	0.18–0.92	0.03

EGJ, esophagogastric junction; irAE, immune-related adverse events; CI, confidence interval; HR, hazard ratio. * Eastern Cooperative Oncology Group performance status. ** Number of treatment regimens from the diagnosis of unresectable advanced or recurrent ESCC until nivolumab. *** At the time of nivolumab initiation. **** Backward elimination method.

## Data Availability

Data presented in this study are available upon request from the corresponding author. The data are not publicly available for further analysis.

## Abbreviations

CI: confidence interval; CR, complete response; CT, computed tomography; DCR, disease control rate; EGJ, esophagogastric junction; ESCC, esophageal squamous cell carcinoma; HR, hazard ratio; ICI, immune checkpoint inhibitor; irAEs, immune-related adverse events; ORR, objective response rate; OS, overall survival; PD, progressive disease; PFS, progression-free survival; PR, partial response; PS, performance status; RECIST, Response Evaluation Criteria in Solid Tumors; SD, stable disease.
